# Disease of the Lungs

**Published:** 1902-02-22

**Authors:** 


### DISEASE OF THE LUNGS.

### TUBERCULOSIS.

**Etiology and Pathology.**—Many valuable and interesting papers have appeared recently bearing on the hotly debated question of the identity of bovine and human tuberculosis. So many distinguished authorities are to be found on either side that, both in Germany and in Great Britain, Royal Commissions have been appointed to re-investigate the whole question. Amongst some of the more important contributions are those by Professor Koch,[1] "The Fight against Tuberculosis in the Light of the Experience that has been Gained in the Successful Combat of other Infectious Diseases;" Professor Hueppe[2] on "Bovine and Human Tuberculosis," advocating the view that they are identical; Professor Cruikshank[3] on "Human and Bovine Tuberculosis;" and Dr. Lydia Rabinowitch[4] on "The Infectiousness of the Milk of Tuberculous Cows," etc., supporting the same views as those of Professor Hueppe; and finally, the papers of Professor Delépine[5] on "The Communicability of Human Tuberculosis to Cattle," and of Mazyck Ravenel[6] on "The Comparative Virulence of the Tubercle-bacillus from Human and Bovine Sources."

*The Personal Factor in Tuberculosis* has, to some extent, been neglected of late, and Sir Dyce Duckworth[7] has done well to reiterate its importance. He says rightly that the *personal factor*, or the relation of the host towards the intruding and infecting parasite, is surely a matter of supreme importance. Duckworth goes on to say that it is not in accord with modern teaching anywhere but in France to discuss or appreciate any particular habits of body or diathetic conditions. These are now regarded as mediæval and effete lucubrations of the older physicians. He cites Osler[8] as stating that "Scrofula is tubercle, as it has been shown that the bacillus of Koch is the essential element," and he remarks that many of our modern pathologists take this view. Duckworth maintains that "there is a class of persons who, by inheritance or acquirement, owing to various debilitating conditions, are frail and delicate, endowed with a specific proclivity to become gravely affected by irritants of all kinds and especially prone to infection by tubercle bacilli. Such persons . . . may never become tuberculous, although always scrofulous." An important question is raised by Maxon King[9] by an analysis of 242 consecutive cases of phthisis, chiefly in private practice, viz., how far tuberculosis in the parents produces hereditary immunity in the offspring. Another well-timed warning is uttered by Kucher,[10] who points out the danger of various prophylactic measures that have been enacted or proposed, and which are all based upon the supposition that the greatest danger in regard to consumption arises from infection. "This is a dangerous belief, as it leads the public to the fallacious conclusion that it is more important to keep away from the danger of infection than to observe the most necessary sanitary laws." He cites Brehmer[11] as saying: "Since 1854 there have been in Gerbersdorf more than 10,000 consumptives. What quantities of sputa have been ejected on either the paths or public highways! What quantities of such sputa, such infectious matter have been carried into the air which has been breathed by the inhabitants of Gerbersdorf! and the percentage of consumptives among them during the last 26 years has decreased from 0·41 to 0·18." King finds that the results in his series of cases show that: (*a*) Of 242 consecutive cases of phthisis, approximately one in every four gave a history of phthisis in the parents. (*b*) Nearly one in three gave a history of previous phthisis in a brother, sister, or both. (*c*) More than two-thirds of those giving a history of previous phthisis in brother, sister, or both had non-phthisical parents. So that, from a study of the foregoing series of cases, the following conclusions naturally follow: 1. The percentage of consumptives having a tuberculous parentage is actually smaller than that having a non-tuberculous parentage, and much smaller than would be more than accounted for by the additional risk of infection to which the former class is subjected. 2. Tuberculosis in the parents renders to no inconsiderable extent an immunity to the disease in the offspring, an immunity which of course is but relative, and not sufficiently protective, but still demonstrable, as is shown by increased resistance to the progress of the disease and increased tendency to recover among this class. In support of his contentions, King remarks that "Within the memory of men still living there was no phthisis among the Indian tribes roaming the high plateaux of Colorado, Arizona, and New Mexico. But a very few years after it became popular to send white consumptives to those regions as a therapeutic measure, the infection thus spread among the Indians gave rise to an almost epidemic form of phthisis of an unusually acute and fatal type. The same is true of the negroes of certain sections of the Southern States, whose heredity was entirely clean so far as tuberculosis is concerned, yet who succumbed in prodigious numbers as soon as the infection was brought them by the consumptive whites from the North, who, by reason of their greater immunity, often recovered by virtue of a climatic influence which, like that of Mentone, was insufficient to protect the non-immune native." The author does not pretend that his series of 242 cases suffice to prove any theory, but at any rate the question he raises is worthy of further consideration and investigation.

**Diagnosis.**—The appearance of a number of venous varicosities, one-third to two-thirds of an inch in length, may often be observed beneath the skin in the neighbourhood of the spines of the seventh cervical and three upper dorsal vertebræ, and is considered by Overend [12] to indicate, or at least to be often associated with, early phthisis. He explains their presence by obstruction to venous circulation from either enlarged and congested bronchial glands in the root of the lung pressing upon the left superior intercostal vein during its passage across the arch of the aorta, or that a thickened pleura may affect injuriously the course of the vein or any of its tributaries and lead to distal engorgement. Overend says it is important to remember that the upper lobes lie tightly fixed within the three upper ribs, and that they reach from the sixth cervical vertebra as far down as the third dorsal spine. [Patients suffering from emphysema, asthma, and bronchitis exhibit varicosities as well, but these are scattered over the chest, both front and back, and along the margins of the lower ribs diffusely and indiscriminately.] When this sign is observed, with the presence of auscultatory signs within the dorsal area corresponding to the apices of the upper and lower lobes, is combined with wasting and myoidema, it is believed by Overend to render the diagnosis of early phthisis, even in the absence of sputum and bacilli, practically conclusive. In contradistinction to this sign of venous varicosities, we may here refer to the "sterno-costal venous festoon" discussed by Thin.[13] The appearance he describes as that of a band of small veins passing downwards and outwards from the lower end of the sternum towards the sides below the nipple line. Frequently this venous festoon stands out boldly on a pale skin on which no other veins are visible, the band being about $\frac{3}{4}$ inch broad. He considers from the position of the festoon that it is composed of venules which unite the two terminal branches of the internal mammary artery, especially the musculo-phrenic, and it is owing to its anatomical position and relation to the diaphragm that it is generally believed to be connected with some abnormality of the pulmonary system. From this view Thin dissents; he believes it occurs in men who occasionally take long-sustained and violent exercise, when their ordinary mode of life prevents them being in "hard" condition. If his hypothesis is correct it shows, says Thin, that the strength of the elements of the venous walls and their power of resistance are closely associated with the "hard" and "soft" condition of the individual.

[1] Brit. Med. Jour., July 27, 1901. [2] Berl. Klin. Woch., Aug. 2, 1901. [3] Lancet, Nov. 2, 1901. [4] Lancet, Sept. 28, 1901. [5] Brit. Med. Jour., Oct. 26, 1901. [6] Lancet, Aug. 17, 1901. [7] Ibid. Nov. 9, 1901. [8] Principles and Practice of Medicine, p. 280. [9] New York Med. Rec., Oct. 12, 1901. [10] Med. Rec., Sept. 28, 1901. [11] Ætiologie der Chronisch. Schwindsucht. [12] Lancet, Aug. 31, 1901. [13] Edin. Med. Jour., Sept. 1901.

